# Identification of Conserved and Divergent Strigolactone Receptors in Sugarcane Reveals a Key Residue Crucial for Plant Branching Control

**DOI:** 10.3389/fpls.2021.747160

**Published:** 2021-11-11

**Authors:** Anqi Hu, Qiaoqiao Zhao, Li Chen, Jinping Zhao, Yuehua Wang, Kuiliang Feng, Ling Wu, Miao Xie, Xuemei Zhou, Langtao Xiao, Zhenhua Ming, Meng Zhang, Ruifeng Yao

**Affiliations:** ^1^State Key Laboratory of Chemo/Biosensing and Chemometrics, Hunan Provincial Key Laboratory of Plant Functional Genomics and Developmental Regulation, College of Biology, Hunan University, Changsha, China; ^2^State Key Laboratory for Conservation and Utilization of Subtropical Agro-Bio Resources, Guangxi Key Laboratory for Sugarcane Biology, College of Life Science and Technology, Guangxi University, Nanning, China; ^3^Hunan Province Key Laboratory of Phytohormones and Growth Development, Southern Regional Collaborative Innovation Center for Grain and Oil Crops in China, Hunan Agricultural University, Changsha, China

**Keywords:** sugarcane, strigolactone, receptor, D14, MAX2, SMXL

## Abstract

Strigolactones (SLs) are a class of important plant hormones mainly regulating plant architecture such as branching, which is crucial for crop yield. It is valuable to study SL signaling pathway and its physiological function in sugarcane, the most important sugar crop, for further molecular breeding. Here, two putative SL receptors SsD14a/b and the interacting F-box protein SsMAX2 were identified in *Saccharum spontaneum*. SL induced both SsD14a and SsD14b to interact with SsMAX2 in yeast. SsD14a, but not SsD14b, could bind with AtMAX2 and AtSMXL7/SsSMXL7. Overexpression of *SsD14a* or *SsMAX2* rescued the increased branching phenotypes of *Arabidopsis thaliana d14-1* or *max2-3* mutants, respectively. Moreover, the crystal structure of N-terminal truncated SsD14a was solved, with an overall structure identical to AtD14 and OsD14 in the open state, consistent with its conserved branching suppression capacity in *Arabidopsis*. In line with the biochemical observations, *SsD14b* could not completely complement in *d14-1* although these two SsD14 proteins have almost identical primary sequences except for very few residues. Complement with the combination of *SsD14b* and *SsMAX2* still failed to rescue the *d14-1 max2-3* double mutant multi-branching phenotype, indicating SsD14b–AtSMXL7 complex formation is required for regulating branching. Mutagenesis analyses revealed that residue R310 at α10 helix of SsD14a was crucial for the binding with SsSMXL7/AtSMXL7 but not SsMAX2. The site-equivalent single-residue P304R substitution enabled SsD14b to bind with AtMAX2 and AtSMXL7/SsSMXL7 and to rescue the phenotype of *d14-1 max2-3* together with SsMAX2. Moreover, this conserved Arg residue across species including rice and *Arabidopsis* determined the activity of SL receptors through maintaining their interaction with SMXL repressors. Taken together, our work identified conserved and divergent strigolactone receptors in sugarcane core SL signaling pathway and revealed a key residue crucial for plant branching control.

## Introduction

Strigolactones (SLs), which function as novel phytohormones in plant branching control (Gomez-Roldan et al., 2008; Umehara et al., 2008), promote the germination of root parasitic weeds (Cook et al., 1966) and regulate the symbiosis of arbuscular mycorrhizal fungi (Akiyama et al., 2005). SL biosynthesis and signaling pathway have become one of the most important and interesting research areas in recent years (Burger and Chory, 2020). Nowadays, enormous efforts have been made in studying SL signaling pathway. Several key components have been characterized, including receptor DWARF14 (D14), F-box protein MORE AXILLARY GROWTH2 (MAX2) and SUPPRESSOR OF MORE AXILLARY GROWTH2-LIKE-6 (SMXL6), SMXL7, and SMXL8 (Stirnberg et al., 2007; Umehara et al., 2008; Arite et al., 2009; Jiang et al., 2013; Stanga et al., 2013; Zhou et al., 2013). Different from other receptors, which could only sense hormone molecules, the receptor D14 have dual roles to sense and hydrolyze SL, demonstrating a brand-new function mode (Nakamura et al., 2013; Jia et al., 2014; de Saint Germain et al., 2016; Yao et al., 2016; Hu et al., 2017; Shabek et al., 2018; Marzec and Brewer, 2019; Seto et al., 2019; Lee et al., 2020). As a bifunctional receptor for SL, D14 is an α/β hydrolase with a complete catalytic triad, S97-H247-D218 (in *Arabidopsis*). The catalytic triad undergoes conformational change and hydrolyzes the four-ring complete SL molecules into two final products containing ABC-ring and D-ring, respectively (Kagiyama et al., 2013; Zhao et al., 2013; Yao et al., 2016; Hamiaux et al., 2018). During the hydrolysis of SL, D14 covalently binds to the D-ring at the catalytic center, then it associates with downstream protein MAX2/D3 to form D14–MAX2/D3 SCF E3 complex. This ubiquitin ligase complex will recruit the downstream transcription repressors SMXL6/7/8/D53, leading to the degradation of SMXLs/D53 through the 26S proteasome pathway (Hamiaux et al., 2012; Jiang et al., 2013; Yao et al., 2016; Wang et al., 2020). Thus, the downstream target genes, such as *Ideal Plant Architecture* 1 (IPA1) (Song et al., 2017) which inhibited by D53, would be released to regulate plant branching. Undoubtedly, the interaction with MAX2 and SMXLs by D14 is the core to turn the transduction system on (Jiang et al., 2013; Zhou et al., 2013; Soundappan et al., 2015; Wang et al., 2015; Yao et al., 2016; Khosla et al., 2020).

As the main sugar crop, sugarcane (*Saccharum* hybrid) has great economic value (Tuma, 1987; Zhang et al., 2012). Modern commercial sugarcane varieties are derived from hybrids between *Saccharum officinarum* L. and *Saccharum spontaneum* L. The yield of *Saccharum* is usually determined by the total number of effective stems and the average single stem weight. Thus, promoting tillering and improving effective tillers are key to increase production (Aitken et al., 2008; Tena et al., 2016; Glassop et al., 2021). As an important parent, *S. spontaneum* is a representative material for sugarcane research, providing the toughness, disease resistance, and regeneration of modern sugarcane, making *S. spontaneum* an important material for SL signaling study.

Here, we studied the function of core SL signaling components from *S. spontaneum*, identified two putative SL receptors with conserved and divergent capabilities to regulate plant branching, respectively, and revealed a key residue crucial for recruiting downstream signaling component and SL responses.

## Materials and Methods

### Generation of Transgenic Plants

The modified vector pCAMBIA1300-cFlag (Yao et al., 2016) carrying the full coding sequence of *Arabidopsis thaliana D14* (*AtD14*), *S. spontaneum D14b* (*SsD14b*), N-terminal (amino acids 1–49) truncated *S. spontaneum D14a* (Ss*D14a*Δ*N*), N-terminal (amino acids 1–44) truncated *S. spontaneum D14b* (Ss*D14b*Δ*N*) and *S. spontaneum MAX2* (*SsMAX2*) under the control of the CaMV 35S promoter was introduced into the *Atd14-1* (Salk_057876) (Waters et al., 2012) or *Atmax2-3* (Salk_092836) (Jia et al., 2014) mutant by using the Agrobacterium-mediated floral dip method.

Similarly, we used GoldenBraid 2.0 system (Addgene^1^) (Sarrion-Perdigones et al., 2013) to construct binary plant expression vectors: 35S:SsD14b–35S:SsMAX2 (P35s:SsD14b:Tnos–P35s:SsMAX2:Tnos–Pnos:NptII:Tnos), 35S:SsD14bP304R–SsMAX2 (P35s:SsD14bP304R:Tnos–P35s:SsMAX2:Tnos–Pnos:NptII:Tnos), and 35S:AtD14–35S:AtMAX2 (P35s:AtD14:Tnos–P35s:AtMAX2:Tnos–Pnos:NptII:Tnos), which were introduced into the *Atd14-1 Atmax2-1 double* mutant, respectively, to generate transgenic plants. The primary rosette branching numbers were counted for 5-week-old plants, which were germinated on plates and grown in soil under a light/dark photoperiod of 16 h/8 h at 22°C.

### Yeast Two-Hybrid Assays

To construct plasmids for yeast two-hybrid (Y2H) assays, the CDS of SsD14a/b and SsD14a/b truncations (SsD14aΔN49 and SsD14bΔN44) were cloned into yeast expression vector pGBKT7 to generate BD-SsD14a/b and BD-SsD14a/b-ΔN, and we also constructed the mutations BD-SsD14aR310P and BD-SsD14bP304R. Similarly, we obtained BD-OsD14 and BD-OsD14ΔN53. The CDS of SsMAX2, AtMAX2, and AtSMXL7 were cloned into pGADT7 to make Gal4 DNA activation domain (AD) constructs, respectively. Y2H assays were performed using the Yeastmaker Yeast Transformation System 2 (Clontech, United States). In brief, yeast strain AH109 cells were co-transformed with specific bait and prey constructs and coating on selective growth medium SD/-Leu/-Trp for 3 days at 30°C, pick the positive constructs into liquid-selective growth medium SD/-Leu/-Trp for 36 h at 30°C, 200 rpm. Washed yeast cells three times and diluted, make sure OD_600_ reached 2.5, then serial 10-fold dilutions of yeast cultures were spotted onto selective growth medium that was supplemented with 5 μM *rac*-GR24 or dimethyl sulfoxide (DMSO). All yeast transformants were grown on selective growth medium at 30°C, 4 days.

### Expression and Purification of SsD14aΔN

The positive clones of *SsD14a*Δ*N* (residues 1–49) proved by DNA sequencing were transformed into *Escherichia coli* strain BL21 (DE3) for protein expression. Kanamycin-resistant colonies were picked to grow in the Luria–Bertani (LB) medium (10 g/L tryptone, 10 g/L NaCl, and 5 g/L yeast extract) at 37°C until OD_600_ reached 0.6–1.0. Then 0.5 mM isopropyl-beta-D-thiogalactopyranoside (IPTG) was added to induce protein expression at 16°C for 18 h. The cell pellet was resuspended in phosphate-buffered saline (PBS) buffer containing 30 mM imidazole, and homogenized by using an ultrahigh pressure cell disrupter (JNBIO, Guangzhou, China). The lysate was centrifuged at 15,000 rpm for 1 h, and soluble proteins were loaded onto the Ni-NTA column. Target proteins were eluted by the PBS buffer containing 300 mM imidazole. The eluted SsD14aΔN (residues 1–49) was further purified by Superdex^TM^75 (GE Healthcare, United States) at 16°C with the buffer containing 150 mM NaCl, 2 mM MgCl_2_, 20 mM Tris pH 8.0, and 10% glycerol.

### Crystallization, Data Collection, and Structure Determination

Purified SsD14aΔN (residues 1–49) (roughly 10 mg/ml) were dissolved in the buffer containing 150 mM NaCl, 2 mM MgCl_2_, 20 mM Tris pH 8.0, and 10% glycerol. The crystals of SsD14aΔN (residues 1–49) were obtained using the hanging-drop method by mixing 1 μl protein with equal volume of reservoir solution containing 0.01 M magnesium chloride hexahydrate, 0.05 M Tris hydrochloride pH 7.5, 5% v/v 2-Propanol at 16°C for 1 week. The data of the SsD14aΔN (residues 1–49) crystal were collected on beamline BL17U1 at Shanghai Synchrotron Radiation Facility (SSRF) and processed by XDS (20124692). The structure of SsD14aΔN (residues 1–49) was determined by molecular replacement, using the structure of OsD14ΔN (residues 1–51) (PDB ID: 3VXK) as the initial searching template. Model building and structural refinement were performed by using COOT (20383002) and PHENIX (22505256), respectively. In the final model, more than 97% residues fall in the favored region in the Ramachandran plot, and the final *R*_*work*_/*R*_*free*_ is 0.1914/0.2275. Data collection and refinement statistics are summarized in Table 1. The atomic coordinates and structure factors have been deposited in the Protein Data Bank.

**TABLE 1:** Data collection and structure refinement statistics.

**Parameters**	**SsD14aΔN**
**Data collection statistics**	
**Cell parameters**	
*a* (Å)	48.81
*b* (Å)	88.29
*c* (Å)	118.52
*α*, *β*, and *γ* (°)	90, 90, and 90
Space group	*P2_1_2_1_2_1_*
Wavelength used (Å)	0.9792
Resolution (Å)	70.81–1.65 (1.74–1.65)
No. of all reflections	356,698
No. of unique reflections	580,49
Completeness (%)	93.6 (99.5)
Average I/σ(I)	12.1 (2.6)
*R*_*merge*_^a^ (%)	11.5 (74.4)
**Refinement statistics**	
No. of reflections used [σ(F) > 0]	110,054
*R*_*work*_^b^ (%)	19.14
*R*_*free*_^b^ (%)	22.75
RMSD bond distance (Å)	0.008
RMSD bond angle (°)	0.909
**Average *B*-value**	
Average *B*-value for protein atoms	28.69
Average *B*-value for solvent atoms	28.61
**No. of atoms**	
No. of protein atoms	415,0
No. of solvent atoms	357
**Ramachandran plot**	
Res. in favored regions (%)	97.94
Res. in outlier regions (%)	0.0

*RMSD, root-mean-square deviations.*

*^*a*^*R*_*merge*_ = Σ*_*h*_*Σ*_*i*_*| *I*_*h,i*_–*I*_*h*_| /Σ*_*h*_*Σ*_*i*_I_*h,i*_*, where, *I*_*h*_ is the mean intensity of the *i* observations of symmetry-related reflections of *h*.*

*^*b*^*R*_*work*_ = Σ(| | *F*_*p*_(obs)| –| *F*_*p*_(calc)| |)/Σ| *F*_*p*_(obs)|; *R*_*free*_ is an *R* factor for a preselected subset (5%) of reflections that was not included in refinement. *F*_*p*_, structure factor of protein.*

*^*c*^Numbers in parentheses are corresponding values for the highest resolution shell.*

## Results

### Identification of D14 Orthologs in *Saccharum spontaneum*

**FIGURE 1:**
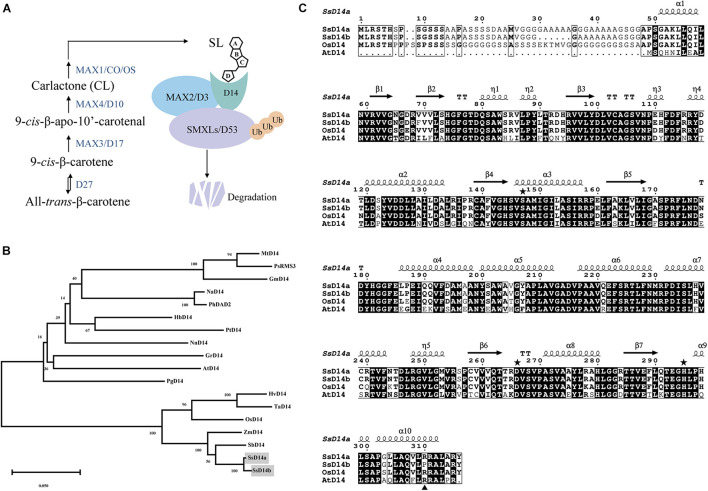
Phylogenetic analysis and sequence alignment of D14 orthologs. **(A)** A simplified model for SL biosynthesis and core signaling pathway. **(B)** Phylogenetic analysis of D14 orthologs. The phylogenetic tree was generated with 18 full-length amino acid sequences of D14 orthologs using MEGA. The evolutionary history was inferred using the Neighbor-Joining method. The percentage of replicate trees in which the associated taxa clustered together in the bootstrap test (1,000 replicates) were shown next to the branches. The evolutionary distances were computed using the p-distance method. Sequence information in this work can be found in GenBank or the Saccharum Genome Database (http://sugarcane.zhangjisenlab.cn/) under the following accession numbers: *Medicago truncatula* D14 (XP_003589086), *Pisum sativum* RMS3 (AMB61024), *Glycine max* D14 (XP_003557012), *Nicotiana attenuata* D14 (XP_019258478), *Petunia hybrida* DAD2 (AFR68698), *Hevea brasiliensis* D14 (XP_021646820), *Populus trichocarpa* D14 (XP_002302409), *Nelumbo nucifera* D14 (XP_010248100), *Gossypium raimondii* D14 (XP_012451974), *Arabidopsis thaliana* D14 (NP_566220), *Punica granatum* D14 (OWM70752), *Hordeum vulgare* D14 (AJP07999), *Triticum aestivum* D14 (AK332360), *Oryza sativa* D14 (XP_015631400), *Zea mays* D14 (NP_001150635), *Sorghum bicolor* D14 (XP_002468316), *Ss5800* (Sspon.001B0005800), and *Ss5830* (Sspon.001B0005830). **(C)** Sequence alignment and structural annotation of D14 orthologs. ESPript was used to analyze the multiple sequence alignments generated by Clustal Omega (Sievers et al., 2011; Robert and Gouet, 2014) with the several D14 orthologs listed in Figure 1A. Secondary structure elements of *Saccharum spontaneum* D14 (GO:0005800) crystal structure (PDB code: 7F5W) are displayed on top of the alignments. Identical and conserved residues are highlighted by red and yellow grounds, respectively. The three catalytic residues, Ser, Asp, and His, are indicated by green stars. The amino acids marked with blue triangles are putative key amino acids for identifying downstream inhibitors.

The SL biosynthesis and core signaling pathways have been thoroughly studied in many plant species including *Arabidopsis thaliana* and *Oryza sativa* (Figure 1A), but remain to be investigated in sugarcane. To identify and investigate the SL receptor(s) D14 in *S. spontaneum* (SsD14), we searched Saccharum Genome Database (SGD)^2^ (Zhang et al., 2018) using BLAST with *Arabidopsis thaliana* D14 (AtD14) and *Oryza sativa* D14 (OsD14) as queries to obtain the predicted sequences of D14 orthologs from *S. spontaneum*. Accordingly, we found two putative *D14* orthologous genes *SsD14a* (Sspon.001B0005800) and *SsD14b* (Sspon.001B0005830) in *S. spontaneum*.

The phylogenetic analysis showed that SsD14s exhibit closer relationships with OsD14 from rice, which belongs to Gramineae too (Figure 1B). The similarity between SsD14a and OsD14 is 84.91% at the amino sequence, and the similarity between SsD14b and OsD14 is 85.94%. Sequence alignment and structural annotation showed that SsD14a/b, AtD14, and OsD14 exhibit both considerable identities at the primary amino acid sequence level and have the same catalytic triad Ser-His-Asp (Figure 1C). These information implies conserved physiological functions of SsD14 proteins.

### SsD14a and SsD14b Have Different Binding Properties With MAX2 and SMXLs

**FIGURE 2:**
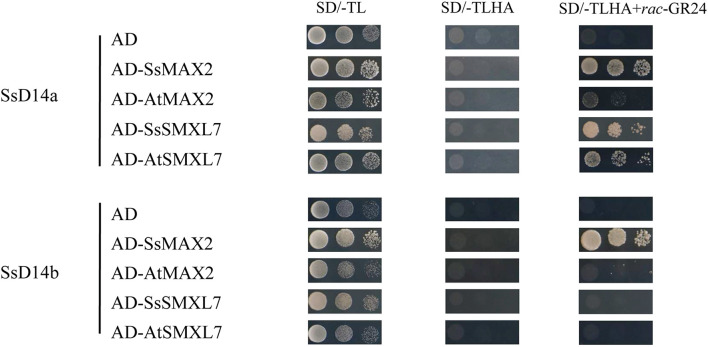
Binding capacity of SsD14s with downstream signaling partners. Yeast two-hybrid (Y2H) assays for SsD14s and AtD14 interactions with SsMAX2/AtMAX2 and SsSMXL7/AtSMXL7. SsD14a, SsD14b, and AtD14 were fused to GAL4-BD. SsMAX2, AtMAX2, SsSMXL7, and AtSMXL7 were fused to GAL4-AD. Serial 10-fold dilutions of yeast cultures were spotted onto the control medium (SD/-Leu/-Trp) and selective medium (SD/-Leu/-Trp/-His/-Ala) in the absence or presence of 5 μM *rac*-GR24 or DMSO control. Images show growth after 4 days at 30°C.

Similarly, we searched SGD to obtain the predicted sequences of MAX2 and SMXL7 orthologs from *S. spontaneum*. Then, we found the putative orthologous genes *SsMAX2* (Sspon.008D0018870) and SsSMXL7 (Sspon.007A0023280). To determine the biochemical function of SsD14a and SsD14b, we used Y2H assays to examine the interaction of SsD14s proteins with SsMAX2, AtMAX2, SsSMXL7, and AtSMXL7. Surprisingly, there were significant binding ability differences between SsD14a and SsD14b. The results showed that SsD14a interacted with SsSMXL7 and AtSMXL7 and interacted with AtMAX2 slightly. However, SsD14b interacted with neither AtMAX2 nor AtSMXL7. Meanwhile, Y2H results showed a strong interaction of SsMAX2 with both SsD14a and SsD14b (Figure 2). In other words, although SsD14a and SsD14b share 97.47% similarity in amino acid sequence, they have different preferences in binding downstream signaling partners, which leading us to speculate that the differences in interactions are attributed to some of these different residues.

### SsD14aΔN, but Not SsD14b and SsD14bΔN, Can Well Rescue the Branching Phenotype of *Arabidopsis d14-1* Mutant

**FIGURE 3:**
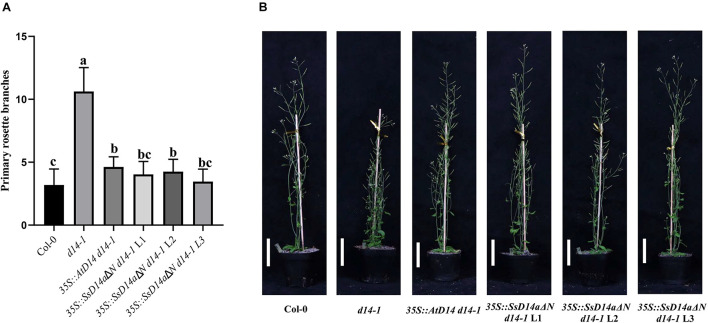
SsD14aΔN can rescue the branching phenotype of *Arabidopsis d14-1* mutant. **(A)** Quantitative analysis on primary branch numbers of Col-0, *d14-1*, *AtD14 14-1*, and three lines for overexpression of *35S:SsD14a*Δ*N*. Values are represented as mean ± SD (*n* ≥ 16); *p* < 0.05 [one-way ANOVA and Tukey’s honestly significant difference (HSD)]. The different letters indicated the different significance. **(B)** The representative branching phenotypes of 5-week-old Col-0 and the indicated mutants. Scale bar = 5 cm.

Previous reports showed that many D14 of Gramineae species contain an extra N-terminal peptides when compared to AtD14 (Yao et al., 2018). Related studies have proved that both the full-length OsD14 and the N-terminally truncated OsD14 were able to complement the multi-branching mutant *Arabidopsis d14-5*, even the N-truncated D14 have more stronger interaction with AtMAX2 and complement *d14* mutant better than the full-length version (Yao et al., 2018). According to our results of Y2H assays, SsD14aΔN can interact with AtMAX2 and AtSMXL7 as the full-length SsD14a did (Supplementary Figure 1). SsD14aΔN was introduced to complement *Arabidopsis d14-1* mutants. We also generated the *35S:AtD14 d14-1* plants as positive control. The results showed no significant difference between the number of primary branches between *35S:SsD14a*Δ*N d14-1* and *35S:AtD14 d14-1* (Figures 3A,B), which means that SsD14aΔN was able to rescue the multi-branching phenotypes. In addition, the leaf morphology (length/width ratio) was also recovered by *SsD14a*Δ*N* (Supplementary Figure 2A). Therefore, SsD14a is functionally conserved when compared with AtD14.

**FIGURE 4:**
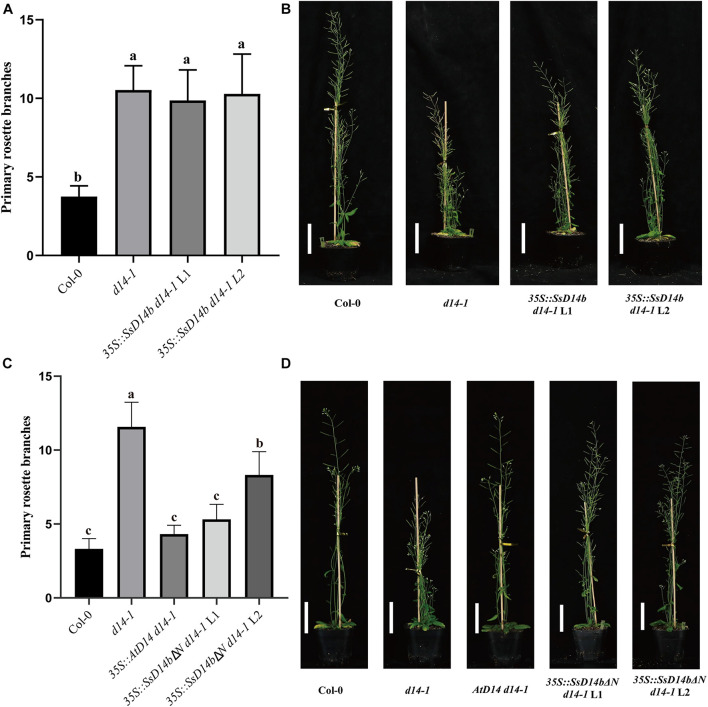
SsD14b and SsD14bΔN failed to well rescue the branching phenotype of *Arabidopsis d14-1* mutant. **(A)** Quantitative analysis on primary branch numbers of Col-0, *d14-1*, and two lines for overexpression of *35S:SsD14b*. Values are represented as mean ± SD (*n* ≥ 16); *p* < 0.05 [ANOVA and Tukey’s honestly significant difference (HSD)]. The different letters indicated the different significance. **(B)** The representative branching phenotypes of 5-week-old Col-0 and the indicated mutants. Scale bar = 5 cm. **(C)** Quantitative analysis on primary branch numbers of Col-0, *d14-1*, *AtD14 14-1*, and two lines for overexpression of *35S:SsD14b*Δ*N49*. Values are represented as mean ± SD (*n* ≥ 16); *p* < 0.05 [ANOVA and Tukey’s honestly significant difference (HSD)]. The different letters indicated the different significance. **(D)** The representative branching phenotypes of 5-week-old Col-0 and the indicated mutants. Scale bar = 5 cm.

However, the complementation results were quite different for SsD14b. According to the Y2H results, neither N-terminal truncated SsD14b nor full-length SsD14b could interact with AtMAX2 and AtSMXL7 (Figure 2 and Supplementary Figure 1). We transferred the full-length *SsD14b* to the *Arabidopsis d14-1* mutant and obtained *35S:SsD14b d14-1* plants. We found that SsD14b cannot rescue the *d14-1* multi-branching phenotype (Figure 4A). But interestingly, we found that the height of transgenic *35S:SsD14b d14-1* seemed to have a partial restoration (Figure 4B), which will be further investigated in the future project. We found that the multi-branched phenotype of one complemented line was only partially restored in *35S:SsD14b*Δ*N d14-1* transgenic lines and still differed from WT, indicating that *35S:SsD14b*Δ*N* cannot fully complement *Atd14-1*. The difference between *35S:SsD14b d14-1* and *35S:SsD14b*Δ*N d14-1* was that multi-branching and the leaf morphology of *35S:SsD14b*Δ*N d14-1* were rescued in different degrees but both not thoroughly (Figure 4 and Supplementary Figure 2B). No obvious interactions of SsD14b/SsD14bΔN with AtSMXLs were detected in our work, which is probably because that the interactions were too weak to be detected in our current Y2H system. Consistent with this, the complementation effect of SsD14bΔN is significantly lower compared to SsD14a. Taken together, SsD14a and SsD14b may function as conserved and divergent SL receptors in sugarcane, respectively.

### Crystal Structure of SsD14aΔN Possesses an Overall Architecture Identical to Other D14 Orthologs in the Open State

**FIGURE 5:**
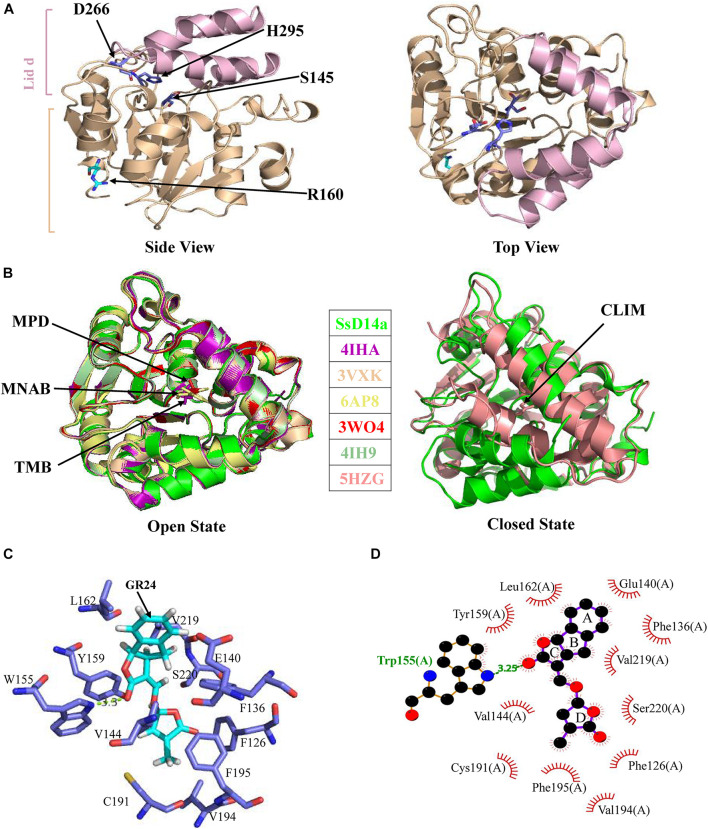
Crystal structure of SsD14aΔN and structural comparisons with other D14 orthologs. **(A)** Crystal structure of SsD14a. Left panel is a side view of SsD14a. The overall structure is represented in cartoon, with the core domain colored in light wheat and the lid domain colored in pink. The three catalytic triad residues are indicated and shown in sticks. Right panel is a top view of SsD14a. **(B)** Structural comparisons reveal that SsD14a is in an open state. Left and right panels show structural comparison of SsD14a with the orthologous proteins of other species in the open and closed state, respectively. AtD14 and OsD14 structures are indicated by their respective PDB IDs, whereas, the structure of SsD14 is indicated by SsD14a. The bound ligands are highlighted and represented as sticks. MPD (in PDB ID 3W04), MNAB (in PDB ID 6AP8), TMB (in PDB ID 4IHA), and CLIM (in PDB ID 5HZG) are abbreviated for 2-methyl-pentanediol, 2-(2′-methyl-3′-nitroanilino) benzoic acid, (2R,3R)-2,4,4-trihydroxy-3-methylbutanal, and (2Z)-2-methylbut-2-ene-1,4-diol, respectively. **(C)** A model for GR24 binding in SsD14a. The SsD14a–GR24 complex is generated by the UCSF DOCK 6.0. Details of GR24 binding are illustrated in the catalytic pocket of SsD14a, which is shown in the light blue cartoon representation. The SL analog GR24, together with its key contacting residues from the binding pocket, are labeled as colored sticks. **(D)** The LigPlot of possible SsD14a–GR24 interactions, related to **(C)**. The red, blue and black atoms denote oxygen, nitrogen and carbon, respectively. Hydrogen bonds between SsD14a and GR24 are shown as green dashed lines. The van der Waals contacts are indicated as continuous red lines.

The crystal structure of SsD14aΔN was determined at a resolution of 1.65 Å (Table 1). SsD14a belongs to the α/β hydrolase superfamily, of which the structure consists of an α/β hydrolase core domain and a four-helix lid domain (αT1, αT2, αT3, and αT4) (Figure 5). The catalytic triad residues of S145, D266, and H295, distributed on the loops following the β4, β6, and β7 strands, are located at the bottom of the hydrophobic substrate-binding pocket. The rest of the core domain is made up of seven β strands (β1–β7) and six α helices (α1, α2, α3, α8, α9, and α10). R310 is located at the α10 helix of SsD14a.

To gain insights into the conformational state of SsD14a, we performed structural comparisons between SsD14a and other D14 orthologs from other plants. Structure comparisons revealed that the overall structure of SsD14a was identical to those from other plants in the open state (Figure 5B), with root-mean-square deviations (RMSD) ranging from 0.250 to 0.301 Å (Figure 5B). Notably, the overall structure of SsD14a in the open state was apparently larger than the closed state of AtD14-CLIM (covalently linked intermediate molecule, a hydrolysis intermediate of SL molecule), thus these two structures cannot be well aligned, with an RMSD of 0.662 Å. Furthermore, results of docking approaches demonstrated extensive binding of GR24 by residues in the catalytic pocket of SsD14a (Figures 5C,D). In general, the structural characteristics of SsD14a are highly conserved and guarantee its branching inhibition function.

### SsMAX2 Rescued the Branching Phenotype of *Arabidopsis max2-3* Mutant

**FIGURE 6:**
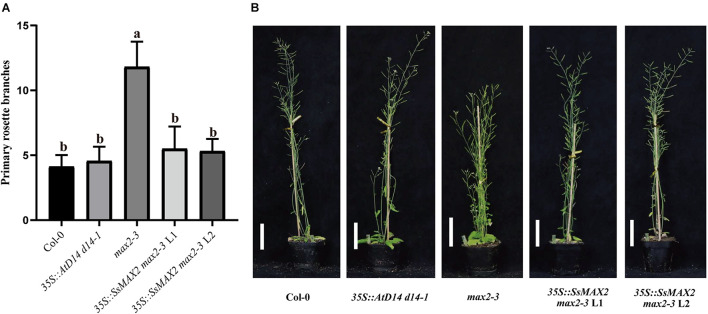
SsMAX2 can rescue the phenotypes of *max2-3* mutant. **(A)** Quantitative analysis on primary branch numbers of Col-0, *max2-3*, *AtD14 d14-1* and two lines for overexpression of *35S:*SsMAX2. Values are represented as mean ± SD (*n* ≥ 16); *p* < 0.05 [ANOVA and Tukey’s honestly significant difference (HSD)]. The different letters indicated the different significance. **(B)** The representative branching phenotypes of 5-week-old Col-0 and the indicated mutants. Scale bar = 5 cm.

To clarify the differences on SL transduction between the two SsD14 proteins, SsMAX2, another key SL signaling transduction component, was obtained and verified its function. SsMAX2 interacted with AtD14 in an SL-dependent manner with the intensity similar to AtMAX2 (Figure 2). We further investigated the physiological function of SsMAX2 proteins in *Arabidopsis*. We introduced full-length *S. spontaneum MAX2* into the *Arabidopsis max2-3* mutant under the control of a 35S promoter. As shown in Figure 6 and Supplementary Figure 2C, *35S:SsMAX2 max2-3* rescued the branching and leaf phenotype of *max2-3* to a level comparable with the wild-type Col-0. These genetic data indicated that SsMAX2 could inhibit axillary branching of *Arabidopsis.* Our results demonstrated that SsMAX2 can resemble AtMAX2 to play a physiological role in *Arabidopsis*.

### Single Residue Substitution of SsD14b Rescues the Binding Affinity With MAX2 and SMXLs

**FIGURE 7:**
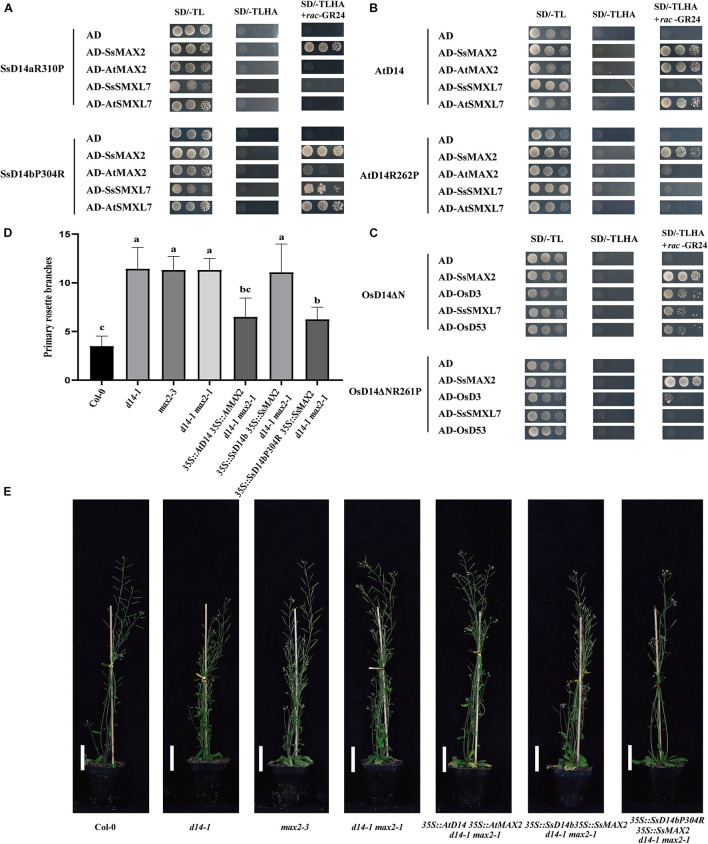
Single residue substitution rescues the biochemical and physiological function of SsD14b. **(A)** Y2H analyses of the interaction between BD-SsD14aR310P and BD-SsD14bP304R for AD-SsMAX2, AD-AtMAX2, AD-SsSMXL7, and AD-AtSMXL7. Serial 10-fold dilutions of yeast cultures were spotted onto the control medium (SD/-Leu/-Trp) and selective medium (SD/-Leu/-Trp/-His/-Ala) in the absence or presence of 5 μM rac-GR24) or DMSO control. Images show growth after 4 days at 30°C. **(B)** Y2H analyses of the AtD14 and AtD14R262P binding with SsMAX2, AtMAX2, SsSMXL7, and AtSMXL7. **(C)** Y2H analyses of the OsD14ΔN and OsD14ΔNR261P binding with SsMAX2, OsD3, SsSMXL7, and OsD53. **(D)** Quantitative analysis on primary branch numbers of Col-0, *d14-1, max2-3*, *d14-1 max2-1*, and T1 lines for overexpression of *35S:D14 35S:MAX2 d14-1 max2-1*, *35S:SsD14b 35S:SsMAX2 d14-1 max2-1*, *35S:SsD14b-P304R 35S:SsMAX2 d14-1 max2-1*, and the indicated mutants. Values are represented as mean ± SD (*n* ≥ 16); *p* < 0.05 [ANOVA and Tukey’s honestly significant difference (HSD)]. The different letters indicated the different significance. **(E)** The representative branching phenotypes of five-week-old Col-0 and the indicated mutants. Bars = 5 cm.

Further sequence comparison with AtD14 and OsD14 found that only SsD14b had a proline (P304) at the α10 helix, whereas, other D14 proteins contained an arginine (R) (Figure 1B). To further explore the mechanism underlying the differences in protein interactions, we made point mutations to SsD14a and SsD14b to obtain BD-SsD14aR310P and BD-SsD14bP304R, respectively. We were surprised to find that the point mutation SsD14aR310P no longer interacted with SsSMXL7 and AtSMXL7 (Figure 7A), but still interacted with SsMAX2. The point mutation SsD14bP304R turn out to obviously interact with AtSMXL7. Inferring from these results, for D14, residue R (like R310 of SsD14a) at the α10 helix might be the key residue contributing to the association with repressor factors SMXLs.

### The R262P/R312P Point Mutation Disrupts the Function of AtD14/OsD14 to Bind With Downstream Signaling Partners

To further investigate the importance and the widespread of the amino acid site of R310 (in SsD14a), we performed point mutation validation in AtD14 and OsD14. We obtained BD-AtD14R262P and BD-OsD14ΔNR261P by site-directed mutagenesis PCR. Y2H results showed that AtD14R262P substitution largely affected the interaction with AtSMXL7 and also greatly weakened the interaction with AtMAX2 (Figure 7B). Similar observation was also found in BD-OsD14ΔNR261P (Figure 7C). Unlike AtD14, OsD14 showed hormone-dependent interaction with SsSMXL7. We speculate that this is a result of the higher sequence similarity between rice and sugarcane, which both belong to Gramineae. Interestingly, AtD14R262P and OsD14ΔNR261P, like wild-type proteins, still have strong hormone-dependent interactions with SsMAX2. The SsMAX2 protein can bind strongly with mutant proteins, which may have application in resolving the crystal structures of certain important D14 mutant proteins in complex with SsMAX2. In general, for AtD14 and OsD14, we further verified the importance of this site for binding downstream signal components.

### Single Residue Substitution Rescues the Physiological Function of SsD14b

In the SL signaling pathway, the D14 receptor senses SL before binding the F-box protein MAX2 to form the D14–MAX2 complex. Later, the complex would recruit and degrade the downstream repressor protein AtSMXLs through ubiquitination–proteasome pathway to regulate plant branching (Jiang et al., 2013; Zhou et al., 2013). To investigate whether SsD14bP304R has gained the capability in plant branching control, we generated and compared the transgenic *Arabidopsis 35S:SsD14b 35S:SsMAX2 d14-1 max2-1* and *35S:SsD14b-P304R 35S:SsMAX2 d14-1 max2-1* by introducing full-length *SsMAX2* together with *SsD14b* or *SsD14b-P304R* into the *d14-1 max2-1* double mutant. We also generated the *35:AtD14 35S:AtMAX2 d14-1 max2-1* plants as positive control. We found that *35S:SsD14b-P304R 35S:SsMAX2 d14-1 max2-1* showed similar primary branching and leaf morphology as *35S:AtD14 35S:AtMAX2 d14-1 max2-1* (Figures 7D,E and Supplementary Figure 2D). However, the complex of SsD14b–SsMAX2 was unable to inhibit the branching of *d14-1 max2-1* double mutant, consistent with the capability of SsD14b or SsD14b-P304R to bind AtSMXL7 (Figures 2, 7A). These results demonstrated that P304R single-residue substitution endows SsD14b with the branching inhibition function, indicating the close correlation between SL responses and receptor–repressor interaction.

## Discussion

Sugarcane is a raw material for sucrose and can also be used as an energy substitute for refined ethanol, which has high economic value. The effective yield of sugarcane is closely related to the effective branching and robust plant architecture. As the ancestor of modern sugarcane and possessing a complete genome database, *S. spontaneum* is an important research material. To lay a foundation for further sugarcane SL pathway studies and related molecular breeding, we turned to identify and study core SL components in *S. spontaneum*.

The SL perception by the receptor D14 initiates the SL signaling transduction pathway. At present, the function of D14 has been studied in many species, such as *Oryza sativa* (D14), *Petunia hybrida* (DAD2), and *Pisum sativum* (RMS3), certificating that D14 is highly conserved in different species (Arite et al., 2009; Hamiaux et al., 2012; de Saint Germain et al., 2016; Yao et al., 2018). Here, two *D14* orthologous genes in *S. spontaneum*, *SsD14a* and *SsD14b*, were identified according to ortholog searching in *S. spontaneum* genome. SsD14a and SsD14b were extremely similar with only few residue exceptions. Additionally, evolutionary analysis showed that both SsD14s were closer to SbD14, ZmD14, and OsD14, all of which are Gramineae. However, Y2H experiments revealed that only SsD14a could interact with AtMAX2 and AtSMXL7/SsSMXL7, whereas, SsD14b could not. Interestingly, there was no difference in the binding affinity with SsMAX2 between SsD14b and SsD14a. Transgenic *Arabidopsis* plants showed that only SsD14a could well rescue the *d14-1* mutant. Furthermore, the structure of SsD14a is identical to AtD14 and OsD14 in the open state, with RMSD ranging from 0.250 to 0.301 Å. These results indicated SsD14a functioned the same as known D14 proteins, such as AtD14, suggesting that a similar SL transduction system exists in *S. spontaneum*.

In the current model, upon perception of SL, the receptor D14 recruits MAX2 and SMXLs to initiate SL signal transduction to regulate branching. However, SsD14b has problems in binding with AtSMXL7/SsSMXL7 and AtMAX2 and is unable to transduce SL signals to inhibit branching by forming such D14–MAX2–SMXL complex. It is interesting that SsD14b, with only very few residue differences from SsD14a, cannot rescue *d14-1* mutant. Meanwhile, through further sequence comparison with AtD14, OsD14, and other reported D14 orthologs, it was found that only SsD14b contains a Proline (P) at position 304, and the rest of the D14 proteins were all Arginine (R) (Figure 1). To verify the effects of this residue site, we obtained point mutations at equivalent sites to obtain SsD14aR310P and SsD14bP304R. After Y2H verification, the point mutation of the two proteins did not affect the interaction with SsMAX2. By contrast, SsD14aR310P no longer interacted with SsSMXL7 or AtSMXL7, but SsD14bP304R interacted with SsSMXL7 and AtSMXL7, suggesting that the R310/P304 site in SsD14s did affect the interaction with the downstream repressor protein SMXLs to form functional D14–MAX2–SMXL complex.

To further verify whether the failure of SsD14b to rescue *Arabidopsis d14-1* is attributed to the loss of SMXL binding ability, we introduced *SsMAX2* together with *SsD14b* or *SsD14b-P304R* into the *d14-1 max2-1* double mutant to express c D14–MAX2 complex. Our results confirmed the importance of SMXL binding by SL receptor and indicated that the assembly of complete D14–MAX2–SMXLs complex is essential for SL responses, although SsD14b–SsMAX2 complex might associate with other proteins but not SMXLs to exert certain function. Additionally, we found that SsMAX2 could bind with D14 proteins from various species much stronger than AtMAX2 and OsD3, suggesting that MAX2 proteins from different plant species may have diverse capabilities to transduce SL signal and would serve as valuable sources for structural studies on SL signaling.

Taken together, our findings shed new light on the study of strigolactone receptors and their interaction with downstream signaling partners, and may have potential application value in the molecular breeding of plant architecture.

## Accession Number

The crystal structure of SsD14a has been deposited in the Protein Data Bank under the accession code 7F5W. *S. spontaneum* genes involved in this article can be found at the Saccharum Genome Database (SGD: http://sugarcane.zhangjisenlab.cn) under the following accession numbers: *SsD14a* (Sspon.001B0005800), *SsD14b* (Sspon.001B0005830), *SsMAX2* (Sspon.008D0018870), and *SsSMXL7* (Sspon.007A0023280).

## Data Availability Statement

The datasets presented in this study can be found in online repositories. The names of the repository/repositories and accession number(s) can be found in the article/Supplementary Material.

## Author Contributions

RY, MZ, and LC conceived and designed the research. AH and MX constructed the vectors. AH performed the yeast two-hybrid assays. QZ and ZM performed the protein purification, crystallization, and structure analysis. AH, JZ, YW, KF, XZ, LW, and XZ conducted *Arabidopsis* transformation and phenotype observations. AH, QZ, LC, LX, MZ, ZM, and RY analyzed the data. AH, QZ, LC, MZ, ZM, and RY wrote the manuscript. All authors read and approved the manuscript.

## Conflict of Interest

The authors declare that the research was conducted in the absence of any commercial or financial relationships that could be construed as a potential conflict of interest.

## Publisher’s Note

All claims expressed in this article are solely those of the authors and do not necessarily represent those of their affiliated organizations, or those of the publisher, the editors and the reviewers. Any product that may be evaluated in this article, or claim that may be made by its manufacturer, is not guaranteed or endorsed by the publisher.

## Footnotes

1
http://www.addgene.org/browse/article/10316/

2
http://sugarcane.zhangjisenlab.cn
