# Supplementary material for: Identification of Conserved and Divergent Strigolactone Receptors in Sugarcane Reveals a Key Residue Crucial for Plant Branching Control
Source: Front Plant Sci. 2021 Nov 11;12:747160. doi: 10.3389/fpls.2021.747160 (PMC8632500; doi:10.3389/fpls.2021.747160)
Supplement: Supplementary file 1 [file Data_Sheet_1.PDF]

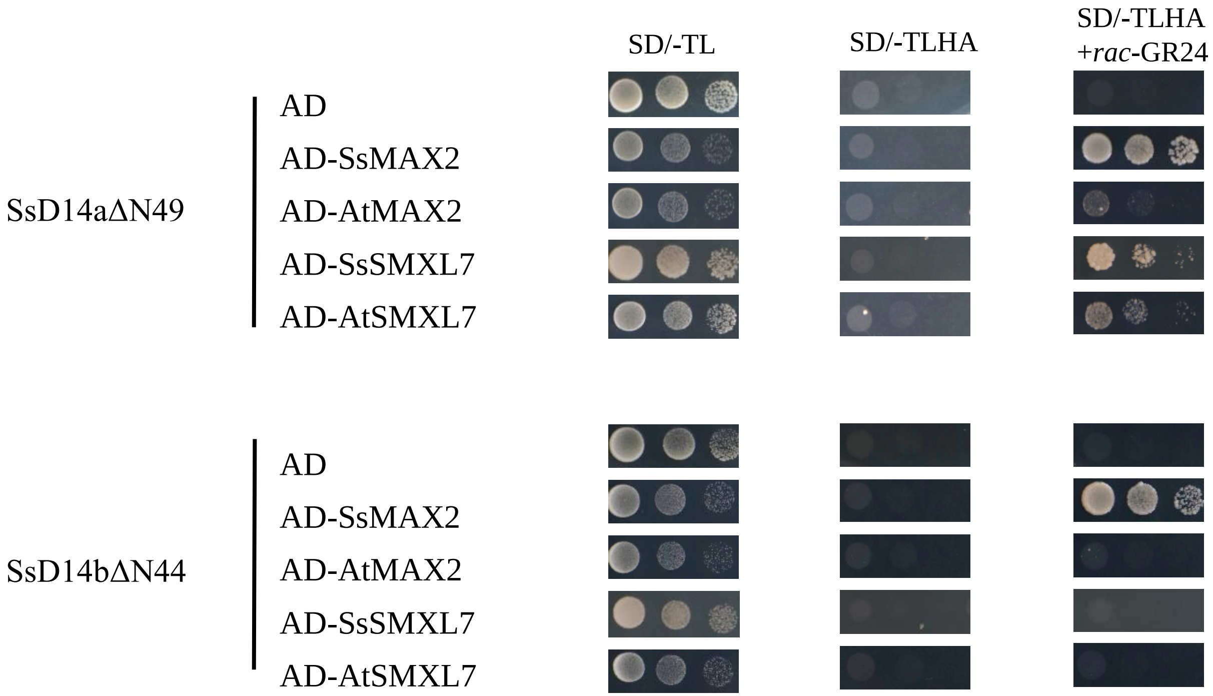

Supplementary Figure1 Binding capacity of SsD14aΔN and SsD14bΔN with downstream signaling partners

SsD14aΔN and SsD14bΔN interact with SsMAX2/AtMAX2 and SsSMXL7/AtSMXL7 in yeast cells upon *rac*-GR24 treatment. SsD14aΔN and SsD14bΔN were fused to GAL4-BD. SsMAX2, AtMAX2, SsSMXL7 and AtSMXL7 were fused to GAL4-AD. Yeast cells were co-transformed with constructs encoding the binding domain (BD) fused to D14 and the activation domain (AD) fused to each MAX2 and SMXL. Yeast transformants were spotted onto the control medium (SD/-Leu/-Trp) and selective medium (SD/-Leu/-Trp/-His/-Ala) in the absence or presence of 5 μM *rac*-GR24. Images show growth after 4 d at 30℃.

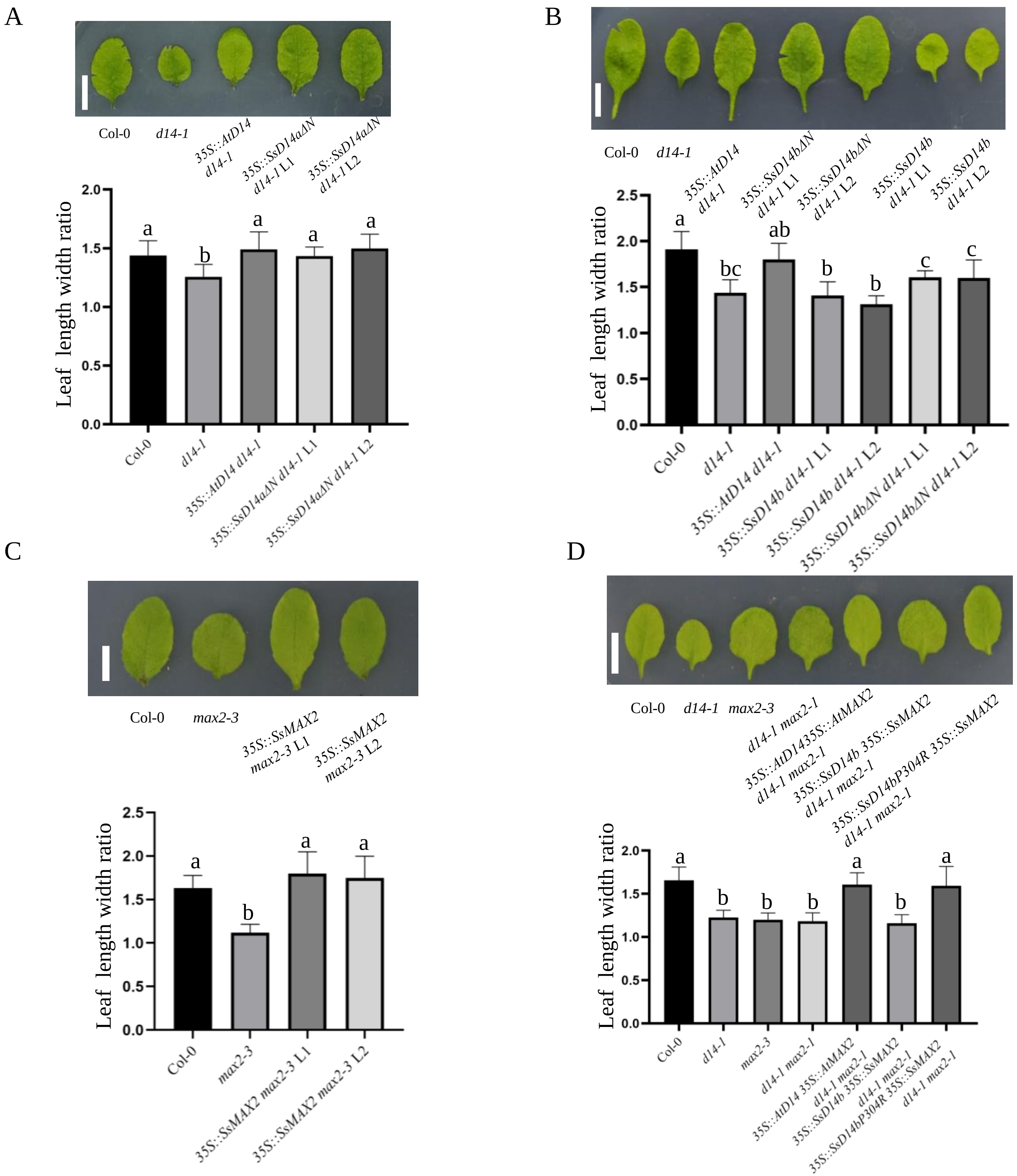
A
Col-0
d14-1
35S::AtD14 d14-1
35S::SsD14aΔN d14-1 L1
35S::SsD14aΔN d14-1 L2
Leaf length width ratio
2.0
1.5
1.0
0.5
0.0
a
b
a
a
a
Col-0
d14-1
35S::AtD14 d14-1
35S::SsD14aΔN d14-1 L1
35S::SsD14aΔN d14-1 L2
B
Col-0
d14-1
35S::AtD14 d14-1
35S::SsD14bΔN d14-1 L1
35S::SsD14bΔN d14-1 L2
35S::SsD14b d14-1 L1
35S::SsD14b d14-1 L2
Leaf length width ratio
2.5
2.0
1.5
1.0
0.5
0.0
a
bc
ab
b
b
c
c
Col-0
d14-1
35S::AtD14 d14-1
35S::SsD14b d14-1 L1
35S::SsD14b d14-1 L2
35S::SsD14bΔN d14-1 L1
35S::SsD14bΔN d14-1 L2
C
Col-0
max2-3
35S::SsMAX2 max2-3 L1
35S::SsMAX2 max2-3 L2
Leaf length width ratio
2.5
2.0
1.5
1.0
0.5
0.0
a
b
a
a
Col-0
max2-3
35S::SsMAX2 max2-3 L1
35S::SsMAX2 max2-3 L2
D
Col-0
d14-1
max2-3
d14-1 max2-1
35S::AtD1435S::AtMAX2 d14-1 max2-1
35S::SsD14b 35S::SsMAX2 d14-1 max2-1
35S::SsD14bP304R 35S::SsMAX2 d14-1 max2-1
Leaf length width ratio
2.0
1.5
1.0
0.5
0.0
a
b
b
b
a
b
a
Col-0
d14-1
max2-3
d14-1 max2-1
35S::AtD14 35S::AtMAX2 d14-1 max2-1
35S::SsD14b 35S::SsMAX2 d14-1 max2-1
35S::SsD14bP304R 35S::SsMAX2 d14-1 max2-1

Supplementary Figure 2 Regulation of leaf morphology by *S. spontaneum* SL-related genes *in Arabidopsis thaliana*. The fifth leaves of 3-week-old wild type (Col-0) and the indicated mutants. Quantitative analysis on the ratio of leaf length to leaf width for the fifth leaves shown in A, B, C and D. Values are represented as mean ± SD ($n \geq 5$); $P < 0.05$ (ANOVA + Tukey (HSD). Bar=1cm.

Supplementary Files The coding sequence (CDS) of SsD14a, SsD14b, SsMAX2 and SsSMXL7.

>SsD14a
ATGCTCCGGTCCACGCACTCACCCAGCGGCAGCAGCTCCGCGGCGCCGGCGTCGTCCAGCTCCGACGCCGCGATGGTCGGGGGCGGCGGC
GCGGCGGCGGCGGCGGGCGGCGGCGCGGCGGCGGCGGCGGGCAGCGGCGGCGCCCCGAGCGGCGCCAAGCTGCTGCAGATCCTCAACGT
GCGGGTCGTGGGCAACGGCGACCGCGTCGTGGTGCTGTCGCACGGGTTCGGGACGGACCAGTCGGCGTGGAGCCGCGTGCTCCCCTACCTC
ACCCGCGACCACCGCGTGGTGCTCTACGACCTCGTCTGCGCCGGCAGCGTCAACCCGGAGCACTTCGACTTCCGACGCTACGACACGCTCG
ACTCCTACGTCGACGACCTCCTCGCCATCCTCGACGCGCTCCGCATCCCGCGATGCGCCTTCGTCGGACACTCCGTCTCAGCCATGATCGGG
ATCCTCGCGTCCATCCGCCGGCCCGAGCTCTTCGCTAAGCTCGTCCTCATCGGCGCATCGCCCAGGTTCCTGAACGACAACGACTACCACGG
CGGGTTCGAGCTGCCGGAGATCCAGCAGGTGTTCGACGCGATGGCGGCCAACTACTCGGCGTGGGCGGTCGGGTACGCCCCGCTGGCGGT
GGGCGCGGACGTGCCCGCGGCGGTGCAGGAGTTCAGCCGCACCCTCTTCAACATGCGGCCGGACATCTCCCTCCACGTCTGCCGCACCGTC
TTCAACACCGACCTCCGCGGCGTGCTGGGCATGGTGCGCTCCCCCTGCGTGGTGGTGCAGACCACCCGCGACGTCTCCGTCCCGGCCTCCGT
CGCCGCCTACCTCAGGGCCCACCTCGGCGGCCGCACCACCGTGGAGTTCCTCCAGACCGAGGGACACCTCCCGCACCTCAGCGCCCCAGGC
CTCCTCGCCCAGGTGCTCCGCCGCGCGCTCGCCCGGTACTAG

>SsD14b
ATGCTCCGGTCCACGCACTCACCCAGCGGCAGCAGCTCCGCGGCGCCGGCGTCGTCCAGCTCCGACGCCGCGATGGTCGGGGGCGGCGGC
GCGGCGGCGGCGGCGGCGGGCAGCGGCGGCGCCCCGAGCGGCGCCAAGCTGCTGCAGATCCTCAACGTGCGGGTCGTGGGCAACGG
CGACCGCTTCGTGGTGCTGTCGCACGGGTTCGGGACGGACCAGTCGGCGTGGAGCCGCGTGCTCCCCTACCTCACCCGCGACCACCGCGTG
GTGCTCTACGACCTCGTCTGCGCCGGCAGCGTCAACCCGGAGCACTTCGACTTCCGACGCTACGACACGCTCGACTCCTACGTCGACGACCT
CCTCGCCATCCTCGACGCGCTCCGCATCCCGCGATGCGCCTTCGTCGGACACTCCGTCTCAGCCATGATCGGGATCCTCGCGTCCATCCGCC
GGCCCGAGCTCTTCGCTAAGCTCGTCCTCATCGGCGCATCGCCCAGGTTCCTGAACGACAACGACTACCACGGCGGGTTCGAGCTGCCGGA
GATCCAGCAGGTGTTCGACGCGATGGCGGCCAACTACTCGGCGTGGGCGGTCGGGTACGCCCCGCTGGCGGTGGGCGCGGACGTGCCCGC
GGCGGTGCAGGAGTTCAGCCGCACCCTCTTCAACATGCGGCCGGACATCTCCCTCCACGTCTGCCGCACCGTCTTCAACACCGACCTCCGC
GGCGTGCTGGGCATGGTGCGCTCCCCCTGCGTGGTGGTGCAGACCACCCGCGACGTCTCCGTCCCGGCCTCCGTCGCCGCCTACCTCAGGG
CCCACCTCGGCGGCCGCACCACCGTGGAGTTCCTCCAGACCGAGGGACACCTCCCGCACCTCAGCGCCCCAGGCCTCCTCGCCCAGGTGCT
TCCGCGCGCGCTCGCCCGGTACTAG

>SsMAX2
ATGGCTGAGGACGCCGCGGCGGGCTCCCCGCTCCTGGACCTGCCGGAGCCGCTGCTGCTCCACATCTTGGGCTTCCTCACCGACGCGCGGTCGCG
GCACCGCGCCGCGCTGGCGTGCCACCGGCTCCTCGCGGCGGAGCGCGCCACGCGCGCCGCGCTCTCGCTGCGCGGGACCCGCGGTCGGACGCG
TTCCTCTACTTCATCAGGCCGACGTTCTGCTTCCCGGCGCTGGAGCGGCTCGACCTCTCGCTGGTGTCGCCGTGGGCCACCCGTTCCTCTCCTAC
GCGGCGCCGTCCGCCGACGCCGTCGCGCCGCCCGTGGCGGCGGAGGAGGTCGCGGGGCAGAACGCCCTCATCGCGGCGCGCCTCGCCTACTGCT
TCCCCGCCGTGTCCTCGCTCGCCGTCTACTGCCGCGACCCCACCACGCTCGCCAGCCTCACCCCGCACTGGAGGTCCCGCCTCAGCAGTGTCAAG
CTCGTGCGCTGGCACCAGCGCCCGCCCGGCCTCGACGCCGGCGCGGATCTCGAGCCGCTCCTCGGGGACTGCCCCGCGCTCAGGGCGCTCGACC
TCTCAGAGTTCTACTGCTGGACGGAGGACATCGAGCCGGCGCTTGCAGCGCACCCTGTAGCCGCCGCGGCGCTCACCGAGCTCGACCTCGGCCT
CGCCGGCGCCACGGACGGGTTCCACGCCACCGAGCTTGGGGCCATCGCGGGATTCTGCCCCAATCTCCGCAAGCTCGTGGCGCCCTGTGTGTTCA
ACCCTCGGTACGTTGATTTCGTCAGCGACGACGCGCTTCTCACCATCGCCACCAGCTGCCCCAAGCTGGCGATCCTGCGGCTCCGGGAACCTTTT
GAGCCGGGGGCTACCGGCCATCGGGAGGACGCGGCAATCACCGTCGCAGGGCTGGTCTCCTTCTTCGCTGCACTACCGGAGCTGGAGGATTTCA
CGCTTGACCTGCGGCATAATGTGCGGGAGACAGCGCCGGCCATGGAGGCGCTTGCCCGCAGATGCCCACGGATCAAGTTCTTGACGCTGGGGGG
CTTCCAGGGGTTGTGCAAGGCATCTTGGCTGCATCTGGACGGCGTTGCTGTGTGCGGTTCGCTGGAGTCTCTATGCATCAAGGGATGTTTGGATC
TCACTGACGCCAGCCTTGTCGCCATAGGTCGTGGGTGCGGGAGGCTAGCTAAGTTCGCGATCCATGGCTGTGATTCTGTCACATCAGCTGGGATC
AGGAGGCTAGCGACGGCGCTTCGGCCCACAATCAAAGAAGTCAGTATCTTGCACTGCCGGCTTCTGGGCACAGCAGCATGCCTCGCTGCTCTAA
GTCCGATCCGTGATCGCATCGAGAGTCTTGAGATCAGCTGTGTCTGGAAGGAAGTTGAACAGCCAGAGAGTGTGGCCAACGGCACAATCGGATG
CGATCATGAAGATGATGATCTCGGTGAAGTGACATATGAGTCTGCATCGAAGAAATGTAGGTACATGGAATTGGATGATCTAGTCAGCTGGGAG
ATGCTACGTTCACTCTCCCTCTGGTTCCCTGCTGGCGAGGTACTCTCCCCACTCATATCTGCGGGCCTTGATAGCTGCCCTGTTCTAGAGGAGATC
TCAATTAAAGTGGAGGGTGATTGCCGTGCACGGCCTGGCCCGTTCTTTGGCCTGAGTGATCTTGCAGGCTTCCCAGTATTAGCCAAGATGAAATT
GGATCTGAGTGAGGCAGTTGGTTATGCTCTTACTGCACCAGCAGGCCAGATGGATCTCTCTCTGTGGGAGCGATTTTATTTGCAAGGTATAGATT
CACTAATGACTTTGTATGAGCTGGATTATTGGCCTCCCCAAGACAAGGAAGTGAACCAGCGGAGCCTGACACTGCCCGCTGTGGGACTGCTCCA
GGGCTGCGTTGGACTCAGGAAGCTCTTCGTCCATGGCACCACACATGAGCATTTCCTGACCTTCTTCTTGAAGGTGCCAAATTTGAGGGACATGC
AGTTACGGGAGGACTACTATCCCGCACCAGAAAGTGATCTGATGAACACGGAAATGCGAGCTGAGTCTTGGCTCCGGTTCGAGAATCAGCTGAA
CATCAGGCTAATTGAGGATTAG

>SsSMXL7
ATGCCGACGCCGGTGCCGCCGCGCGGCAGTGCCTCGCCCGGCCGCCGTCACGGCCCTCGACGCCGCCGTCGCGTCCGCGCGCCGCCGGGCGCACGCGCAGACCACCTCC
CTCCACCTCATCGCCTCGCTCCTGTCCCCACCACCGCGCCGCTGCTCCGCGACGCGCTCGCCCGCGCCCGCAGCTCCGCCTACTCCCCGCGCCTCCAGCTCAAGGCGCTCGA
CCTCTGCTTCGCTGTCTCCCTCGACCGCCTCCCCTCCACCCCCACCTCTTCCGTCTCCGCCTCTGCCACCACCAGCAGCAGCAATGACCAGCACGAGCCCCCCGTCGCCAACT
CCCTCATGGCCGCCATCAAGCGCTCGCAGGCCAACCAGCGCCGCAACCCGGACACGTTCCACTTCTACCCCCACCACCACCAGCAGCAGGCGGCGGGCGCGTCGGCCACCT
CGCCCAACGCTGTCAAGGTCGACCTTTCCCACCTCGTGCTCGCCATCCTCGACGACCCGCTCGTCAGCCGCGTCTTCGCCGACGCCGGGTTCCGCAGCAACGAGATCAAGGT
CGCCATCCTCCGCCCCGCGCCGCCCGTCCCGCTGCTCGGCCGCGGCCTCCCCCTCCCCACGCGCGCCCGCCCTGCGCCGCTCTTCCTCTGCAGCTTCGCTGCCGCCGACGACG
CCGACGTCCCCTCGCCCGCGCCTGCCCTCGCAGGGGCCGCCCCGGGGGAGGACAACTGCCGCCGCATCACCGACATCCTCGCCCGCGGACGCAACCCCATGCTCGTCGGCG
TCGGGGCCGCGTCCGCCGCCGCCGACTTCGCCAACGCGTCACCGTACCGCATCCTCCCCGTCAATCATCAAACGGACCTCCTCGCCGTCGCGGCGGCACCGACGACGCCCGG
TTCTGGCCTCATCTTCAGCATCGGTGACCTCAAGGACCTGGTGCCCGACGAGGCCGACCTGCAGGACGCGGCTCGCCGGGTGGTGGCGGAGGTCACGCGCCTGCTCGAGAC
GCACAGAGCTGCCGGCCGCCAGACGGTCTGGGTCATGGGCTGGTCGGCCACCTACGAGACCTACCTCGCCTTCCTTTCCAAGTTCCCGCTCGTCGACAAGGACTGGGAACTC
CAGCTGCTGCCAATCACCGCCGTGCGCGACGCCGGCCCTGCGGCCGGACTCGTGCCTCCTCCAGCTCCAGCCACCACGGTCGGTGCCTTGTCCATGCCTGCCACTACAAGCT
TCATGGAGTTGTTTGTCCCTTTTGGAGGTTTTATGTGCAATACCTATGAAGCAAGTAGCCTCACAGCAAATTCCTGCCCTCAGGCCCTGCGATGTCAACAGTGCAACGATAG
ATATGAGCAAGAAGCTGCAACTATCATTAGAGGAAGTGGCATTACAGCTGAAGCTCACCAAGAAGGTCTACCTTCCCTGGTGCAGAATAGCAGCATGTTGGGTCCTAACAA
TGGGTTTGATGCAATCAAGGTTAGAGATGATCAGATGGTATTGAGTACAAAAATACAGAATCTGAAGAAGAAGTGGAATGAGTACTGCCTACGACTCCACCAAGGTTGCAA
TAGGACCAACAGAGATCCTTGCCAGTTATTTCGACATCACATCGATGTTCGAGTTGACAGGGAAAGATGTGCAAATCCAAACCAAAGTTCACAGTCAGTTGCACTTCAAAG
GGAGGTTATTAGACCTTCCGCAGTGTCTTCTCCACATACCAACACAACTGCAAAGAGTATTTCGGCGCCATCTATTTCCACCCAAATGAATGCAGACCTTGTATTGAACCTTC
AAGTCAGGCAATCAAAGAGTGATGAACCCCTCCAAGATAGGGCTGTGCCATCCCAACATAGCAACTCATCAAATTGTGACAACCCTGAAGATCATGTGTCACCATCATCTG
CTGCACCTGTGGCCACTGACTTGGTGTTGGCCACCCCTCGTGGATCTTCTTCCAAGGATTCAAGTAATGCACTGTGTAAACATGTAGAGGATGCTGAAGGGTCGATCCAGCT
GATGCCGAAGAAGGTTGATGATCTGAATCGTAAGCCTCCTCACTTCTCTGCACAGCCTTACACTTGCTTCAGGAGTTCCTCAAATTGGGATCAAACTTCACCTAGTGCTCTGC
ATTCAGCAGCTTCAGGAGGTGCTTCTGCCTTTGACCAGTGGCAGAGGCCTTCACCCCTCGCAGCACAAAGTTATGATTTGAGCAATTACAAGCTACTCATGGAACGACTGTT
TAAGGCTGTTGGGAGGCAGGAGGAAGCCTTGAGTGCTATTTGTGCATCCATTGTGCAGTGCAGGTCAATGGAGAGGCGTCGTGGTGCAAGCAAGAAGAATGACATATGGTT
TAGTTTTTATGGCCCGGATGGCATTGCCAAGCGGAGAGTTGGTGTGGCACTTGCTGAGCTAATGCATGGTAGCTCAGAGAACCTGATATATCTGGACCTAAGCCTCCATGAT
TGGGATAACCCCAATTTCAGAGGAAAGCGTGCCACCGACTGTATCGCTGAAGAGTTGAGAAAGAAGCGGCGATCAGTTATCTTCCTTGACAATATCGAGAAAGCTGACTGC
CTTGTTCAGGAGAGCCTGATTCATGCCATGGAGACTGGCAGGTACAAAGACTTGCATGGGGGACGGGAGGCTGACCTTAATGACTCGATTGTGGTGTTGTCTACAAGAATG
AACCAAGGATGTCAGGATGCTTCTCTTGGGATGGAAGAGGGCAATGCTTTTTCAGAAGAAAAGGTTGTGGCAGTTCGTGGAAATCAACTGAAGATCATAGTTGAACCAAGC
ACAGCCAATATCAGTGGAGGCCCTGGAGGCAAAGTTGTAGTTTCCTCAAGGCATTCCTCGATAAACAGTCAAGCATCTTTGTACTCCAGTTCTTTCAGTAAGCGGAAGCTCC
ACATCTCTGATGGGCAAGAAAAGACATCACAATCACCGAGCACTTCAAAGCGACTGCATAGAACATCAAGTGTTCCATTCGACTTGAACCTCCCAGGCGACGAGGCTGAAG
CCCAGGATGGTGATGACGACAGCAGTAGCAGCCATGAGAACTCATCCAGTGATCCAGAGGGATCTTTGGGCAGCCTCTTACGATCAGTGGACGAGTCAATCAATTTCAAGC
CATTTGACTTTGGCAAACTTTGTGAGGACATCCTGCAGGAGTTTAGCAGTACTATGAGCAAGACTCTGGGCTCCAGGTGCAGGCTGGAGATCGACGCTGTGGCTATGGAGCA
AGTAGTGGCAGCGGCATGGGCATTCGATTCACATGAGAAGAGGCCTGTGCGGACGTGGGTGGAGCAGGTGTTTGCCAGAAGCCTTGAGCAGCTGAAGGTCAGGTGTAAGA
ATTTGAGCAGCTGTACACTGAGATTGGTTGCCTGTGAGGATGAAACGCCGGTGAAAGAAGACGGTTTTCTTCCCTCGAGAATAATTCTGGATTGGTGA
